# A unified resource for transcriptional regulation in *Escherichia coli* K-12 incorporating high-throughput-generated binding data into RegulonDB version 10.0

**DOI:** 10.1186/s12915-018-0555-y

**Published:** 2018-08-16

**Authors:** Alberto Santos-Zavaleta, Mishael Sánchez-Pérez, Heladia Salgado, David A. Velázquez-Ramírez, Socorro Gama-Castro, Víctor H. Tierrafría, Stephen J. W. Busby, Patricia Aquino, Xin Fang, Bernhard O. Palsson, James E. Galagan, Julio Collado-Vides

**Affiliations:** 1 Centro de Ciencias Genómicas, Universidad Nacional Autónoma de México, Cuernavaca, Morelos México; 2 School of Biosciences, University of Birmingham, Birmingham, UK; 3 Department of Biomedical Engineering, Boston University, Boston, Massachusetts USA; 4 Department of Bioengineering, University of California San Diego, La Jolla, California USA; 5 Center for Biosustainability, Technical University of Denmark, Kongens Lyngby, Denmark

**Keywords:** Transcriptional regulation, Transcriptomics, Integrative analyses, Systems biology, ChIP-seq, gSELEX

## Abstract

**Background:**

Our understanding of the regulation of gene expression has benefited from the availability of high-throughput technologies that interrogate the whole genome for the binding of specific transcription factors and gene expression profiles. In the case of widely used model organisms, such as *Escherichia coli* K-12, the new knowledge gained from these approaches needs to be integrated with the legacy of accumulated knowledge from genetic and molecular biology experiments conducted in the pre-genomic era in order to attain the deepest level of understanding possible based on the available data.

**Results:**

In this paper, we describe an expansion of RegulonDB, the database containing the rich legacy of decades of classic molecular biology experiments supporting what we know about gene regulation and operon organization in *E*. *coli* K-12, to include the genome-wide dataset collections from 32 ChIP and 19 gSELEX publications, in addition to around 60 genome-wide expression profiles relevant to the functional significance of these datasets and used in their curation. Three essential features for the integration of this information coming from different methodological approaches are: first, a controlled vocabulary within an ontology for precisely defining growth conditions; second, the criteria to separate elements with enough evidence to consider them involved in gene regulation from isolated transcription factor binding sites without such support; and third, an expanded computational model supporting this knowledge. Altogether, this constitutes the basis for adequately gathering and enabling the comparisons and integration needed to manage and access such wealth of knowledge.

**Conclusions:**

This version 10.0 of RegulonDB is a first step toward what should become the unifying access point for current and future knowledge on gene regulation in *E*. *coli* K-12. Furthermore, this model platform and associated methodologies and criteria can be emulated for gathering knowledge on other microbial organisms.

## Background

Similar to the role that the elucidation of the structure of DNA had in the foundation of modern genetics, the concepts more recently revealed about transcription factor binding sites (TFBSs) and their effects on the activity of promoters that transcribe transcription units, operons, and regulons serve as the foundation for how we think about gene regulation in microbial organisms, and with some modifications, in higher organisms as well. These concepts were the product of research in *Escherichia coli* K-12 during the second half of the twentieth century. They underlie the computational infrastructures for electronic databases on microbes, such as RegulonDB, to encode and populate all knowledge that molecular biologists have generated, from the time of the seminal works by Jacob and Monod to today. Over 20 years of continued curation have resulted in the placement of every binding site, promoter, transcription factor (TF) and its active conformation, or any other piece of published knowledge on gene regulation, in their corresponding coordinates of the updated complete genome sequence of this bacterium.

**Fig. 1:**
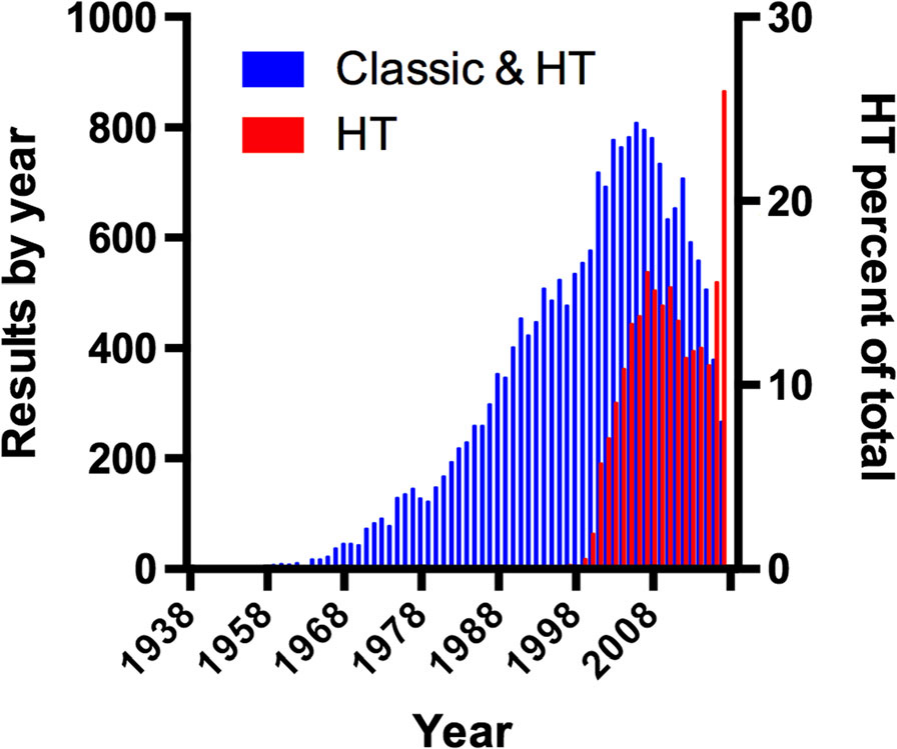
Number of publications studying transcriptional regulation in *E*. *coli* K-12, using either classic molecular biology or HT technologies through the years

However, the emergence of “postgenomic methodologies” has changed the game. We now have whole-genome expression profiles for thousands of different conditions (e.g., the COLOMBOS and M3D databases [1, 2]) and whole-genome identification of binding sites for around 65 TFs; these numbers continue to increase. During the last decade, we have seen a sharp increase in the number of studies on transcriptional regulation in *E*. *coli* K-12 involving different high-throughput (HT) approaches (Fig. 1), and it is likely that we are transitioning to high-throughput (HT) approaches dominating research, as opposed to the more directed molecular biology experiments already deposited in RegulonDB. See the variety of novel HT methodologies shown in Table 1.

**Table 1:** Search results by methods

Method	PubMed search	Datasets
ChIP-chip	30	34
ChIP-seq	15	34
ChIP-exo	8	12
Selex	35	23
RNA-seq	160	102
Microarrays	1188	749
Hi-C	8	1
IPOD	1	1
NET-seq	2	2
TraDIS	3	0
ChAP-seq	2	0
CLIP-seq	1	1
Bisulfite-seq	1	0
Genotyping	n.d.	10
RIP-seq	n.d.	2
Others	0	157
Total	1454	1128

In the midst of the accelerated pace of generation of data and experimental information in the genomic era, databases and other electronic resources are the major instruments with which to integrate and facilitate access to the tsunami of data otherwise only incompletely captured by individual investigators. Table 2 lists the major databases and repositories with information about the biology of *E*. *coli* K-12. The two up-to-date manually curated databases are RegulonDB [3] and EcoCyc [4]**.** Our team is in charge of curating transcriptional regulation for these two databases. On the other hand, COLOMBOS is the only database with microarray data specific for *E*. *coli*, and it also contains similar data for a few other microorganisms [1]. Otherwise, HT data are found in the general repositories GEO and ArrayExpress (Table 1).

**Table 2:** Resources for gene regulation in *E. coli* K-12

Source	Type of knowledge	URL	Updated	Reference
RegulonDB	Transcriptional regulation, operons, regulons, gensor units	http://regulondb.ccg.unam.mx	Yes	[3]
EcoCyc	Regulation, transport, metabolism	https://ecocyc.org	Yes	[4]
COLOMBOS	Expression compendia of bacterial organisms	http://colombos.net	Yes	[1]
STRING	Protein-protein interaction network	http://string-db.org	Yes	[20]
GEO	Genomics HT data repository	https://www.ncbi.nlm.nih.gov/geo/	Yes	[21]
ArrayExpress	Repository of HT functional genomics experimental results	https://www.ebi.ac.uk/arrayexpress/	Yes	[22]
PortEco	Next-generation data for *Escherichia coli*	http://porteco.org	No	[5]
GenExpDB	Expression compendia	https://genexpdb.okstate.edu	No	–
EcoGene	*E*. *coli* K-12 genome and proteome information	http://ecogene.org	No	[23]
GenProtEC	Functions encoded by the *Escherichia coli* K-12 genome	http://genprotec.mbl.edu	No	[24]
EchoBASE	Information from postgenomic experiments	https://www.york.ac.uk/res/thomas/	No	[25]
Bacteriome	Integrates physical (protein-protein) and functional interactions	http://www.compsysbio.org/bacteriome/	–	[26]
EcoProDB	Integrates protein information	http://eecoli.kaist.ac.kr/main.html	–	[27]
M3D	Resource for microbial gene expression data	http://m3d.mssm.edu	No	[2]

Years ago, there were efforts in the USA to organize HT data for *E*. *coli*. These included EcoliHub and its subsequent PortEco version, in addition to EcoliWiki; none of these is currently actively maintained [5]. Therefore, an investigator interested in gathering what is currently known about a particular regulatory system in *E*. *coli* has to spend time searching these different resources.

Given that HT methodologies enrich our knowledge on gene regulation and gene expression, expanding the current model beyond RegulonDB is a natural next step. However, this is not a straightforward task. HT data sometimes challenge the Jacob and Monod paradigm, such as when there is supporting evidence for a binding site far from any promoter, or when a promoter site is found in a non-coding region between two convergent ends of genes, where no transcription initiation is expected to occur. HT methodologies generate large amounts of what sometimes appears as disconnected pieces of data. For instance, a single study might reveal ≈ 14,000 candidate transcription start sites (TSSs), of which more than 11,000 occur within the coding regions (≈ 5500 in the sense strand and ≈ 5400 in the antisense strand) [6]. Similarly, it is no longer surprising to find binding sites within the coding regions in HT binding experiments. The number of these TSSs or binding sites that are either non-functional or that participate in roles not directly related to gene regulation is still an open question.

As a result, we need a mixed model that can accommodate both the complete picture of a transcription unit with its promoter and binding sites where objects and their interactions make sense, as well as plausible but disconnected objects. First, the data should be available in a structured way when possible, but with enough flexibility to allow users to make their own decisions. Second, we need to implement tools and criteria to identify experiments performed under similar conditions. An ontology and its corresponding controlled vocabulary for precisely defining growth conditions are part of our efforts in this direction [7]. This is the basis for merging our classic curation with the one presented here for HT binding experiments, together with the expression profiles to identify the effects of binding, to construct a regulatory interaction. Third, we need to define additional evidence codes for different types of HT experiments, together with the limits that define when there is sufficient information to include a new regulatory interaction or any other piece of evidence that contributes to plausible regulatory processes, as opposed to scattered elements without enough support for their interpretation as functional elements of gene regulation. Finally, we have to define the features of and how to display HT-generated binding sites and regulatory interactions in a way consistent with those that already exist. Altogether, this constitutes the basis for adequately gathering and enabling the comparisons and integration needed to manage the vast current knowledge about transcriptional regulation in *E*. *coli*. We present here the first version of a more complete integration of HT binding experimental results (from chromatin immunoprecipitation [ChIP] experiments and genomic systematic evolution of ligands by exponential enrichment [gSELEX] data) with the previously curated literature.

## Methods

### Search of literature and datasets involving HT technologies

A literature search was focused in PubMed. We collected publications involving the HT methodologies shown in Table 1. Searches were performed looking for the term “coli” in the title or in the abstract and the name of the method or different synonyms or keywords related to the method in all fields of publications. This strategy usually resulted in repeated studies; thus, we filtered the unique results. We read the abstracts and discarded all those papers not reporting experiments explicitly performed in *E*. *coli*. Finally, we filtered once again the repeated studies among all considered techniques, obtaining 1454 unique studies (Table 1 and Fig. 1).

**Fig. 2:**
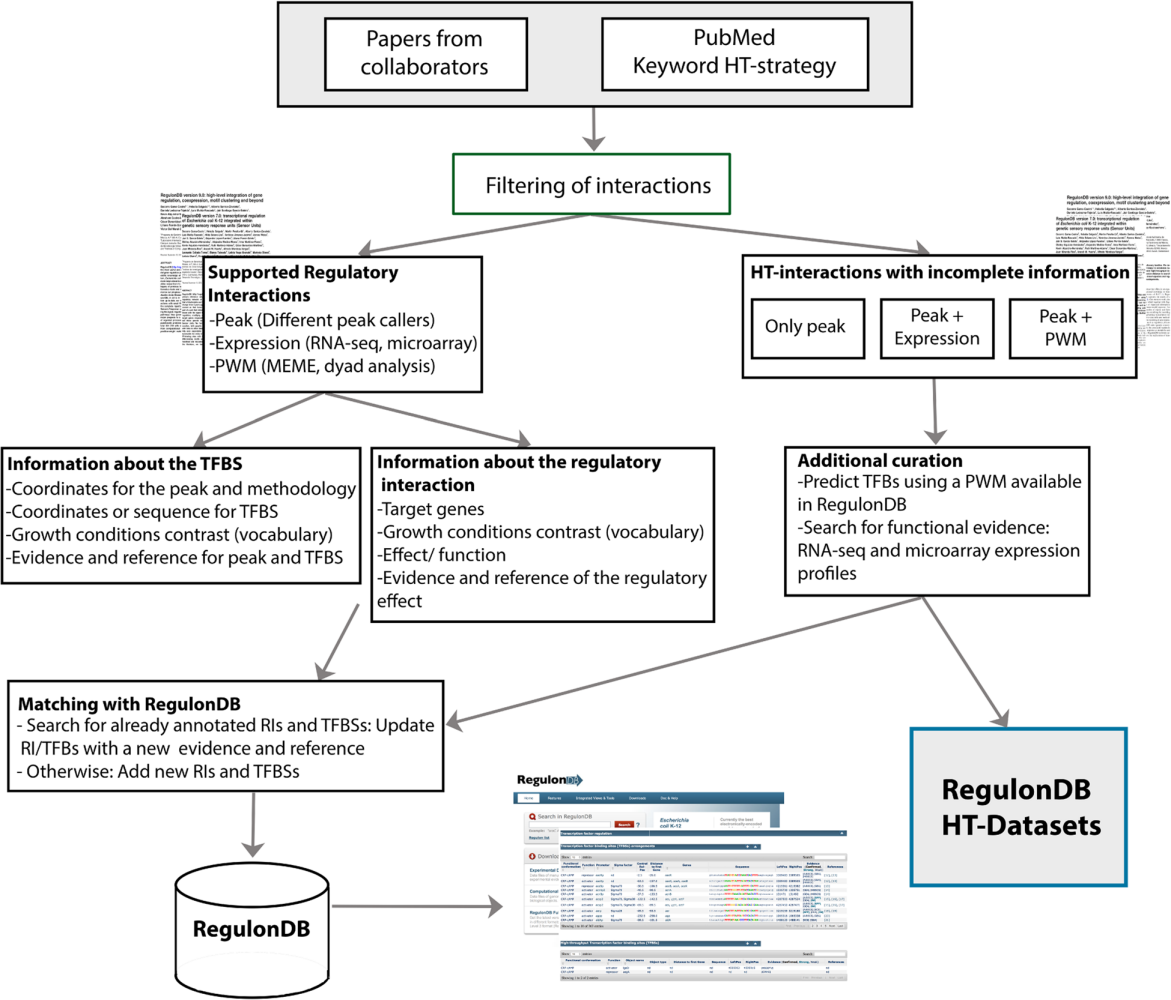
Diagram of the annotation process. We collect publications in PubMed involving the HT methodologies. Searches were made for the term “coli” in the title or in the abstract and the name of the method or different synonyms or keywords related to the method in all fields of publications. The results were filtered to get unique results. We read the abstracts and eliminate all those papers not reporting experiments performed in *E. coli*. Frequently, the papers include additional experimental characterization for a subset of the sites based on classic methods. Metadata are extracted from each publication. For more detail see main text. The growth condition and strain are described using the controlled vocabulary defined by Tierrafría et al. [7]. The products of curation are added to RegulonDB either together with the classic curation or as a separate dataset. Image from RegulonDB [8]

Of the 1454 papers related to HT, 1188 belong to microarrays, leaving only 248 papers related to ChIP-X (either ChIP-seq, ChIP-exo, or ChIP-chip), gSELEX, and RNA-seq, in addition to 18 papers with a variety of HT techniques (see Table 1). Fortunately, essentially all microarray datasets are incorporated in the COLOMBOS database. Fifty-one papers were processed in order to extract all peak sequences or regions identified by HT methods. Frequently, these papers include additional experimental characterization for a subset of sites based on the results of electrophoretic mobility shift assays, footprinting analysis, and bioinformatics tools, primarily via the use of position weight matrices (PWMs) for the TFBSs to precisely identify the binding sites in the sequences of the peak regions. Curation of the literature extracted from each publication included the following metadata: the strain; growth condition; number of targets; name of the TF; methodology used ChIP-X, gSELEX, or RNA-seq and its evidence code; additional techniques used to further identify the binding sites; and links to the files, when available, in the repositories of GEO or ArrayExpress. As mentioned above, the growth condition and strain are described using the controlled vocabulary defined by Tierrafría et al. preprint [7]. As explained in the section on the curation of HT literature, the products of curation are added to RegulonDB either together with the classic curation or as a separate dataset. For those added to the classic curation, the information includes on the one hand information about the binding such as coordinates for the peak and methodology, coordinates or sequence for TFBS, growth conditions, evidence and reference, and information about the regulatory interaction: target genes, methodology, growth condition contrast (frequently comparing expression of overexpressed TF vs its mutant knockout), effect or function, evidence, and reference of the regulatory effect. In the case of regulatory interactions identified in the *E*. *coli* K-12 substr. W3110, we verified that the TFBS sequence is conserved with *E*. *coli* K-12 substr. MG1655, before adding it in RegulonDB. A schematic of the overall flux of our process is shown in Fig. 2, also found under in the annotation process under about RegulonDB [8].

We also indicate if the effect was identified by the authors (with their corresponding thresholds of change of expression), and we specify the regulated gene. Information on peak sequences is contained in the datasets. It is important to keep in mind that once the DNA sequences identified by a particular antibody are sequenced, these are then mapped to the genome sequence, and the sequence peaks or regions are defined; in these experiments, these regions are usually in the range of 200 to 500 nucleotides. We refer to them as peak sequences. A subsequent step is the identification of potential precise binding sites for the given TF. Most often, this is currently done via alternative bioinformatics methods that use known PWMs within those regions, such as MEME [9] or dyad analysis or other similar methods [10], although alternative methods also exist [11, 12]. We gather information on the method used by the authors, as well as the evidence according to the notation used in RegulonDB, which expands that reported by the Gene Ontology Consortium, see the page of evidence classification on Regulon DB [8].

In several cases, the sequences that result from the peak-calling algorithms were provided without identification of a precise binding site. In those cases, the curator team used the PWM available in RegulonDB (under external data, in the matrix alignments [8]) for the given TF to search among the peak sequences by using the threshold parameters adequate for each TF. The selection of the threshold was decided using the score distribution matrix [13] using the separation between the empirical and theoretical distribution. All data for TF motif matrix are available on RegulonDB in the matrix alignment page [8].

## Results

This paper is focused on the literature from HT binding experiments. Our curation focused on identifying the objects (sites, promoters, interactions) that satisfy a set of criteria regarding confidence and interpretability (see below), in order to upload them in RegulonDB together with all existing knowledge. When these criteria are not satisfied, then we simply offer the data as datasets (searching for downloads [8]), which are not equally browsable or displayed within RegulonDB, as explained below. We curated a total of 51 papers with HT approaches out of which we added 1048 new regulatory interactions of 9 TFs, in addition to 107 existing regulatory interactions that have been found by these methods. These papers generated 16,609 interactions of 36 TFs and sigma factors that have some missing information and therefore are included only as datasets. Note that we distinguish regulatory interactions from plain “interactions,” for which no evidence is yet available supporting their regulatory role.

### Curation of HT literature in RegulonDB

As reported in our publications describing our progress with RegulonDB, we have curated some papers from past HT experiments. The first datasets we included were for TSSs identified by Illumina sequencing of 5′-triphosphate-enriched transcripts by the group of Morett [14]. In 2015, we initiated the curation of binding sites obtained via gSELEX (CRP, H-NS, and LeuO) and ChIP-exo (GadE, GadW, GadY, OxyR, and SoxS), as well as the dataset of TSSs reported by the group of Storz [6]. We are now including curated sites and have made a separate section so that the user can easily identify the datasets coming from HT experiments, together and/or separated from those coming from classic methods. Furthermore, we have initiated important modifications to the computational model of RegulonDB, together with a controlled vocabulary for growth conditions which, taken together, prepare us for a constant and eventual up-to-date curation of all of this literature’s content. We have extracted publicly available information for 43 different TFs from experiments performed in *E*. *coli* K-12 by ChIP (ChIP-chip, ChIP-seq, and ChIP-exo) or gSELEX by the group of Ishihama; their experiments were performed in *E*. *coli* strain K-12 sub-strain W3110 [15] (this is noted in RegulonDB), as well as RNA-seq and microarray information contained in those papers. Curation of this literature included extracting the metadata (see the “Methods” section) that contain all relevant information of the biology (TF and growth conditions) as well as links to the data if found in standard repositories, and also relevant information as detailed in the “Methods” section. A total of 51 new papers were curated of which 19 are papers with gSELEX data, 17 from ChIP-chip data, 8 from ChIP-seq data, and 7 from ChIP-exo data. The summary of all curated knowledge from HT methodologies currently available in RegulonDB is shown in Table 3. While this is an important first step, additional data are continually curated in order to reach an up-to-date level equal to that of the classic literature.

**Table 3:** Summary of all curated knowledge available in RegulonDB that was obtained via HT methodologies

Methodologies	Number of articles	Number of TFs	Name of the TFs
gSELEX	2, previous work	3	CRP, H-NS, and LeuO
	19, this work	18	AscG, BasR, CitB, Cra, CsgD, Dan, DpiA, LeuO, Lrp, NemR, OmpR, PdhR, PgrR, RcdA, RstA, RutR, SdiA, and SutR
ChIP-chip	1, previous work	1	PurR
	17, this work	15	ArcA, ArgR, CRP, Fis, FNR, H-NS, IHF, LexA, Lrp, NsrR, RpoD (Sigma70), RpoH (Sigma32), RutR, Rho, and TrpR
ChIP-exo	2, previous work	6	GadE, GadW, GadX, OxyR, SoxS, and SoxR
	7, this work	4	ArgR, Fur, OmpR, and UvrY
ChIP-seq	8, this work	8	CsiR, FNR, Fur, H-NS, Nac, OmpR, RpoD (Sigma70), and RpoS (Sigma38)
Methodologies	Number of articles	Number of TSSs	Dataset in RegulonDB
TSS determination	2, previous work	5197	http://regulondb.ccg.unam.mx/menu/download/high_throughput_datasets/ [8]
		1806	http://regulondb.ccg.unam.mx/menu/download/high_throughput_datasets/ [8]
	1, previous work	14000	http://regulondb.ccg.unam.mx/menu/download/high_throughput_datasets/ [8]

### Criteria to combine classic and HT-supported data

When curating knowledge on gene regulation in *E*. *coli*, the best decision we can make is to offer users the best possible integration of data and information, clearly indicating the corresponding experimental method and reference. The challenge of the classic paradigm of gene regulation with the scattered data from HT experiments is solved in practice by separating two sets as the product of our curation: those pieces of knowledge (TFBSs) with enough additional evidence to support their functional role in gene regulation are added to the bulk of existing knowledge (see Table 4), whereas those binding sites for which not enough information is known about the bound TF and its role in gene regulation are kept in separate datasets (see Table 5). Additionally, experiments kept in datasets are those that support a given DNA region in the genome that is usually much larger than TFBSs, such as peak regions or regions from SELEX experiments, but for which a precise TFBS has not been identified.

**Table 4:** Summary of curated HT-generated regulatory interactions. The total of new RIs is 1048 and those RIs already existing are 107

Complete data uploaded in RegulonDB							
	Datasets		Regulatory interactions				
TF	Total number of peaks	Sites with missing information	New	Known (added in evidence)	PMID	HT methodology	Reference
ArgR	122	37	67	18	25735747	ChIP-exo, qPCR, and microarray	[28]
ArgR	48	34	10	4	22082910, this work	ChIP-chip and microarray	[29], this work
ArcA	278	143	115	20	24699140	ChIP-chip, qPCR, and microarray	[30]
CsiR	126	0	126	0	28061857	ChIP-seq and RNA-seq	[16]
FNR	224	186	29	9	24699140	ChIP-chip, qPCR, and microarray	[30]
FNR	53	0	29	24	23818864	ChIP-seq and microarray	[31]
Fur	144	87	39	18	25222563	ChIP-exo and RNA-seq	[32]
Fur	134	119	12	3	26670385, this work	ChIP-seq and microarray	[33], this work
Lrp	143	67	68	8	19052235	ChIP-chip and microarray	[34]
Nac	534	0	531	3	28061857	ChIP-seq and RNA-seq	[16]
OmpR	41	31	10	0	26332955	gSELEX	[35]
OmpR	41	30	11	0	28526842	ChIP-exo and RNA-seq	[36]
TrpR	8	7	1	0	22082910, this work	ChIP-chip and microarray	[29], this work

**Table 5:** Summary of interactions curated in datasets

TF interactions within datasets				
TF	Number of interactions	PMID	HT methodology	Reference
ArcA	143	24699140	ChIP-chip	[30]
ArgR	426	22082910	ChIP-chip	[29]
ArgR	38	25735747	ChIP-exo	[28]
AscG	9	19633077	gSELEX	[37]
BasR	99	22442305	gSELEX	[38]
CitB	15	18997424	gSELEX	[39]
Cra	14	16115199	gSELEX	[40]
Cra	234	21115656	gSELEX	[41]
CRP	39	16301522	ChIP-chip	[42]
CsgD	31	21421764	gSELEX	[43]
CsiR	126	28061857	ChIP-seq	[16]
Dan	176	20156994	gSELEX	[44]
DpiA	15	18997424	gSELEX	[39]
Fis	228	16963779	ChIP-chip	[45]
FNR	137	17164287	ChIP-chip	[46]
FNR	796	23818864	ChIP-seq and ChIp-chip	[31]
FNR	186	24699140	ChIP-chip	[30]
Fur	473	26670385	ChIP-seq	[33]
Fur	91	25222563	ChIP-exo	[32]
H-NS	1501	23818864	ChIP-chip	[31]
H-NS	101	16963779	ChIP-chip	[45]
H-NS	53	21097887	ChIP-seq	[47]
IHF	1020	23818864	ChIP-chip	[31]
IHF	155	16963779	ChIP-chip	[45]
LeuO	17	19429622	gSELEX	[48]
LexA	69	16264194	ChIP-chip	[49]
Lrp	67	19052235	ChIP-chip	[34]
Lrp	296	28348809	gSELEX	[50]
Nac	537	28061857	ChIP-seq	[16]
NemR	6	18567656	gSELEX	[51]
NsrR	83	19656291	ChIP-chip	[52]
OmpR	68	28061857	ChIP-seq	[16]
OmpR	30	28526842	ChIP-exo	[36]
OmpR	31	26332955	gSELEX	[35]
PdhR	14	17513468	gSELEX	[53]
PgrR	82	23301696	gSELEX	[54]
RcdA	39	23233451	gSELEX	[55]
RstA	34	17468243	gSELEX	[56]
RutR	20	18515344	ChIP-chip	[57]
RutR	9	17919280	gSELEX	[58]
SdiA	212	24645791	gSELEX	[59]
SutR	15	25406449	gSELEX	[60]
TrpR	17	22082910	ChIP-chip	[29]
UvrY	288	26673755	CHIP-exo	[61]
Sigma factors and Rho interactions within datasets				
Sigma factors and Rho	Number of interactions	PMID	HT methodology	Reference
RpoD (Sigma70)	1214	16109958	ChIP-chip	[62]
RpoD (Sigma70)	528	16301522	ChIP-chip	[42]
Rho	260	19706412	ChIP-chip	[63]
RpoD (Sigma70)	6350	23818864	ChiP-seq	[31]
RpoH (Sigma32)	82	16892065	ChIP-chip	[64]
RpoH (Sigma32)	44	20602746	ChIP-chip	[65]
RpoS (Sigma38)	91	26020590	ChiP-seq	[66]

Users can download and combine the information available within the classic model of RegulonDB with any of the available datasets, and we plan on implementing additional tools in the future that will facilitate their comparison, visualization, and processing. As these tools are implemented, the decision as to what information gets added to the core of knowledge and what remains as datasets will be less relevant in practice.

Our curation strategy involves two phases. First, we curate all of what is reported in a single paper. We start by identifying all those binding sites showing evidence of a role in gene regulation, including additional experiments reported to strengthen selected cases. In the second phase, we search in other publications and datasets in order to find evidence needed to suggest effects on regulation, activation, or repression of transcription for additional binding sites. We specifically combine data from gene expression generated by RNA-seq and/or microarray experiments with data from TF DNA-binding experiments. To do so, we use our parallel work of mapping growth conditions in RegulonDB with growth conditions reported in COLOMBOS. Such a mapping and definition of a controlled vocabulary is an enormous task that is ongoing, but in our coordinated work, we have made sure that the conditions present in our meta-curation for HT experiments are included, for details, see Tierrafría et al. preprint [7].

The central question then is what is the minimal evidence that supports a site found to have a functional role in gene regulation, based on either any ChIP type of experiment (ChIP-seq, ChIP-exo, or ChIP-chip) or by gSELEX. First, the binding site sequence has to be identified; otherwise, the TF target gene could be an indirect target. The stronger cases are those with a sequence identified for binding of a TF, frequently identified by a computational search in the peak sequence, and the effect on regulation suggested by an observed change in gene expression. We assign the effect (activator, repressor, or dual effect) determined for the regulated gene or transcription unit. If the regulatory interaction and TFBS are not already present in RegulonDB, this information is added as a new site and a new regulatory interaction. If the data already exist in the database, then the new evidence is added to the existing regulatory interaction(s) (Table 4).

In cases where the authors have not identified the precise TFBS, we use the PWMs in RegulonDB and search for a binding site in the sequence, and only when a site is found, the information is added as a regulatory interaction.

The following cases are considered to have insufficient information to conclude whether they play a role in gene regulation. We exclude those where a binding site is identified but has no evidence with which to assign an effect and a regulated gene. In other cases, the corresponding expression experiment has been performed but there is no evidence of change in expression of the downstream gene. Some possible reasons for this could be an inactive conformation of the TF or coregulation missing under the conditions studied, or the protein effectively binds but has no role in transcriptional regulation. Furthermore, for now, we have decided that peak sequences with or without a binding site that fall in regions of the genome where no transcription is expected, such as within a coding region or within a convergent region surrounded by the ends of two genes, are not further analyzed; information for such sequences can be accessed only as datasets. We are aware that additional work can be done, for instance, by searching for nearby TSSs, curating antisense transcription (currently available in datasets), and curating cases of TFBSs within genes with a regulatory effect (see the site for Nac inside the *gadE* gene and Tables 3 and 4 in Aquino et al. [16]).

In addition to the evidence code and the method, our classification of evidence is reported as either confirmed, strong, or weak. Evidence codes come from the Gene Ontology Consortium, which is shared in our curation of both RegulonDB and EcoCyc. In order to facilitate the processing of the diversity of evidence codes by the user, in RegulonDB, we describe them in three classes: “confirmed” when they have more than one independent solid evidence, “strong” for cases supported by physical evidence, and “weak” in other cases (such as a computational prediction). Objects with multiple independent weak evidence entries are upgraded to strong. A detailed explanation for this process is found on the evidence classification page in RegulonDB [8], which was the subject of reference [17]. Note that we always include the precise evidence codes for added detail, in the event that users do not like the classification of types of evidence unique to RegulonDB. A summary of the results of this curation is shown in Tables 4 and 5. We call HT-supported regulatory interactions those sites that satisfy the minimal criteria outlined, and HT binding sites are those left as datasets.

### Display in RegulonDB

All these curated HT-supported regulatory interactions are now present within RegulonDB version 10.0 and can be found on the regulon page of the corresponding TF. The most direct way to access them is to type the TF name followed by “regulon,” go to the link of the regulon, and display the TF regulon page. On that page, there is a table with all TFBSs, which now includes those derived from HT experiments. Table 4 describes all TFs with HT-supported regulatory interactions in the current version of the database. Furthermore, via the “Downloads” main page menu, HT datasets and any of the TF-specific HT binding datasets can be selected. Both of them (individual HT-supported TFBSs and specific datasets) can be browsed by searching for growth conditions, for example, using their contrasting experimental vs control condition change(s). Additionally, as already mentioned, a search using the controlled vocabulary for growth conditions will show both the structured data as well as the link to the datasets. We are working to display any dataset as a track in our browser, which will enable the direct comparison with, for instance, information coming from classic experiments and with any other annotations available in RegulonDB.

## Discussion

As mentioned above, we do not want to dilute the predominantly high-confidence knowledge that has come from classic experimental methods, aimed at identifying individual objects or interactions, with the massive but more fragmented knowledge that HT methodologies produce [3], which by its nature involves several layers of experimental treatments and subsequent processing by bioinformatics and statistical methods. Thus, not only do experimental methodologies vary but also the bioinformatics programs and the selection of thresholds used in the different processing steps vary. Nonetheless, as shown in Fig. 1, the tendency of the literature is the continuous and more dominant use of HT-based methods in research, which has led to the urgent need for the expansion of RegulonDB we have described here. This requires the modification of several components of our system, starting with a computational model with a more precise encoding of the distinct, almost elementary components that constitute the knowledge of gene regulation. We now require evidence, methods, and reference for the binding site of a given TF and for its effect on a regulated gene or promoter, and we need to indicate the expression profile experiment that supported a change in expression of the (candidate) regulated gene. We also distinguish which piece of information comes from the literature and which one comes from our own active curation. It is important to note that even classic experiments generate, by the nature of the experimental work, pieces of evidence that are gradually constructed to generate a more or less complete picture. For instance, the gene regulated by a TF is frequently identified by transcriptional constructions with a reporter gene. Strictly speaking, this evidence only supports the fact that RNAP proceeds into transcription downstream of the promoter; whether it transcribes in vivo, only the first downstream gene or the complete transcription unit requires identification of such a transcript under precisely the same control and regulated conditions.

**Fig. 3:**
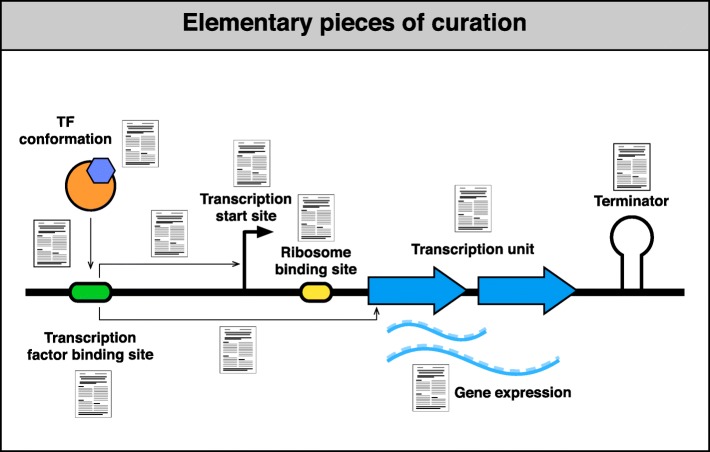
Elementary pieces of curation. As new methods emerge, we need to separately curate evidence and references for each elementary piece of knowledge that, when combined, supports our understanding. Here, we have separated evidence for binding of TFs and evidence for an effect on transcription either of a known promoter or on a target gene or TU for which the promoter is not known

Our controlled vocabulary and collection of features, generically called “growth conditions,” also contribute to higher precision, by annotating the strain or genetic background used in the experiment as well as growth conditions minimally required for their replicability. We believe that as we advance in this deconstruction to the “elementary pieces of knowledge” from experiments (Fig. 3), we will be better prepared to incorporate experimental findings obtained via new methodologies that will continue to emerge in the future. This expanded model affects the internal structure, the tools for curation, and the display for users to access the data. In this paper, we have focused essentially on HT alternatives that identify binding sites for transcriptional regulators at a genomic level. These experiments identify the bound sites in the genome, some of which may have a role in vivo affecting gene regulation, but others may have no role at all affecting transcription, and therefore, even the name “transcription factor binding sites” may be misleading in those cases.

The strategy used both in the computational model and in the display of knowledge enables users to decide if they want to see either the knowledge that comes from molecular biology experiments, that from HT-based methods, or from both types.

## Conclusions

We consider the work presented here to be a first version of what we envision will be a long-term project to integrate the different elements involved in gene regulation. Certainly, there is plenty of room for improvements. Many more analyses can be implemented in cross-comparisons of the increasing volume of HT datasets, so that new correlations may emerge. In this sense, the curation presented here has only used the assignment of the effect of TFBSs by searching the biologically adequate expression profile (the comparable growth condition and strain) to see if a change of expression of the downstream gene was observed. In fact, many more analyses can be performed. For instance, it will be useful to offer datasets that provide partial knowledge regarding the regulation of gene expression by unknown mechanisms, such as those occurring within coding regions [16]. Additional programs need to be implemented to search for all binding sites if there are TSSs found nearby, including the thousands present in our datasets. The relative distance between a TFBS and its regulated TSS is known to be correlated with the activating or repressing function [18, 19]; some sigma factors are associated with particular conditions, like stress or heat shock. All of this information (and more) provide seeding for pipelines to be implemented for a more automatic and periodic update in the generation of evidence for gene regulation. This suggests a new type of “bioinformatics biocuration,” a more active process gathering evidence across multiple publications and experiments to reconstruct the different elements and interactions required for our understanding of the regulation of transcription initiation and to distinguish those elements involved in gene regulation by unknown mechanisms as well as those that may have different roles associated with their binding in yet unknown processes in evolution.

## Abbreviations

ChIP: Chromatin immunoprecipitation
gSELEX: Genomic systematic evolution of ligands by exponential enrichment
HT: High-throughput
PWMs: Position weight matrices
TF: Transcription factor
TFBSs: Transcription factor binding sites
TSSs: Transcription start sites
TU: Transcription unit
